# Two new species of the Andean genus *Xenophyllum* (Senecioneae, Compositae)

**DOI:** 10.3897/phytokeys.139.47872

**Published:** 2020-01-27

**Authors:** Joel Calvo, Vicki A. Funk

**Affiliations:** 1 Instituto de Geografía, Facultad de Ciencias del Mar y Geografía, Pontificia Universidad Católica de Valparaíso, Avenida Brasil 2241, 2362807 Valparaíso, Chile Pontificia Universidad Católica de Valparaíso Valparaíso Chile; 2 US National Herbarium, Department of Botany, Smithsonian Institution, Washington D.C. USA Smithsonian Institution Washington United States of America

**Keywords:** Andes, Argentina, Asteraceae, Ecuador, taxonomy, *Werneria*

## Abstract

Two new species of *Xenophyllum* are described from the Andes, *X.funkianum***sp. nov.** from Ecuador and *X.lorochaqui***sp. nov.** from northwestern Argentina. Both species are compared with the morphologically closest taxa and useful characters for their proper identification are provided. Detailed illustrations and distribution maps are also presented, as well as pictures of living plants when available.

## Introduction

The Andean genus *Xenophyllum* V.A.Funk (Senecioneae) was coined to embrace 21 species that were traditionally placed under the Neotropical genus *Werneria* Kunth (Weddell 1856; Blake 1928; Rockhausen 1939; Cabrera 1948). Funk (1997) transferred to *Xenophyllum* those species developing genuine or rhizome-like stems and narrowed the *Werneria* concept to the species strictly rosettiform or scapiform. Such circumscription was later adopted by most botanists working on this plant group (e.g., Nordenstam 1999; Freire and Ariza-Espinar 2014; Jørgensen 2014; Beltrán 2016; Calvo et al. 2017).

The species belonging to *Xenophyllum* are strictly Andean, distributed from northeastern Colombia to northern Argentina and Chile. They are suffruticose plants displaying involucral bracts fused at the base, capitula radiate (disciform in one species), ray florets usually white (yellow or pink in a few species), filament collar balusterform, and style branches truncate with a crown of sweeping hairs or penicillate. Two main subgroups can be differentiated according to the type of growth form. One includes the species forming dense mats or hummocks, which have rhizome-like stems with marcescent leaves along them. The other subgroup embraces those species with genuine erect stems forming clumps or even developing a shrubby habit.

Ongoing studies aiming to obtain a modern and comprehensive taxonomic revision of the genus *Xenophyllum* led us to describe two new species, one from Ecuador and the other from Argentina. The Ecuadorian species has a restricted distribution area nearby the Chimborazo Volcano and is known from a few gatherings. The Argentinian species thrives in the northwestern provinces and was widely confused with other members of the genus. Detailed descriptions, illustrations, distribution maps, and taxonomic discussions are provided for each species, as well as pictures of living plants when available.

## Materials and methods

This contribution is the result of an intensive review of the published bibliography and the revision of herbarium specimens kept at BA, BM, BR, GH, LIL, QCA, QCNE, US, and W. Additionally, a digital herbarium specimen was obtained from SI; herbarium acronyms follow Thiers (2019+). A Standard 16WL microscope was used for the examination of the achene trichomes. Fieldwork was conducted in Ecuador.

## Taxonomy

### 1. Xenophyllum funkianum

Taxon classificationPlantaeAsteralesAsteraceae

J.Calvo sp. nov.

48DAAAA6-64BA-5A53-8C39-6BD97874AADE

urn:lsid:ipni.org:names:77204849-1

Figs 1 , 2

#### Diagnosis.

*Xenophyllumfunkianum* is well characterized by its creeping rhizome-like stems 20–35 cm long, the straight linear leaves prolonged into a sheath-like base that bears arachnoid trichomes, the dark-burgundy sessile involucres with 13–14 involucral bracts, the 12–13 white ray florets somewhat purplish beneath, and by having white-villous achenes.

#### Type.

**Ecuador.** Chimborazo: Mt. Chimborazo area, at the end of *Polylepis* road and beginning of hike to *Polylepis* forest, 1°31'50"S, 78°52'55"W, 4233 m, 20 Apr 2018, *V.A. Funk & J.M. Bonifacino 14059* (holotype: US!; isotypes: MO!, QCA!).

#### Description.

Suffruticose plants, forming creeping lax mats, with rhizome-like stems 20–35 cm long, 0.3–0.5 cm in diam., covered by arachnoid indumentum and leaf-base remnants resembling paleae, horizontal, simple or branched from the base. ***Stems*** 2–3 cm long (aerial part), arachnoid. ***Leaves*** simple, alternate, imbricate, straight, prolonged into a sheath-like base that bears arachnoid trichomes; leaf laminas linear, 5.3–7.8 mm long, 0.8–0.9 mm wide, entire, rather acute, callous-tipped at the apex, elliptical in cross section, glabrous, unconspicuously nerved above, 1-nerved beneath (only conspicuous on the lower third), fleshy, shiny, papillose. ***Capitula*** radiate, solitary, terminal, erect, sessile. ***Involucres*** 9–11 mm long, 5–7 mm wide, cupuliform, with bracts fused at the base, glabrous; involucral bracts 13–14, 4.7–6.9 mm long, 1.0–1.7 mm wide at the base, acute at the apex, dark-burgundy; without supplementary bracts. ***Ray florets*** 12–13, corollas 8.9–11.6 mm long, 2.3–3.0 mm wide, 4-veined, subentire to 3-toothed at the apex, conspicuously surpassing the involucre, white, somewhat purplish beneath. ***Disc florets*** 20–23, corollas 5.0–5.6 mm long, 5-lobed, yellowish; style branches truncate with a crown of sweeping hairs, yellowish; anther bases obtuse; anther appendages two times longer than wide, ca. 0.4 × 0.2 mm. ***Achenes*** cylindrical, with white-villous trichomes (immature); pappus 3.9–6.2 mm long, barbellate, whitish. Chromosome number unknown.

**Figure 1.**
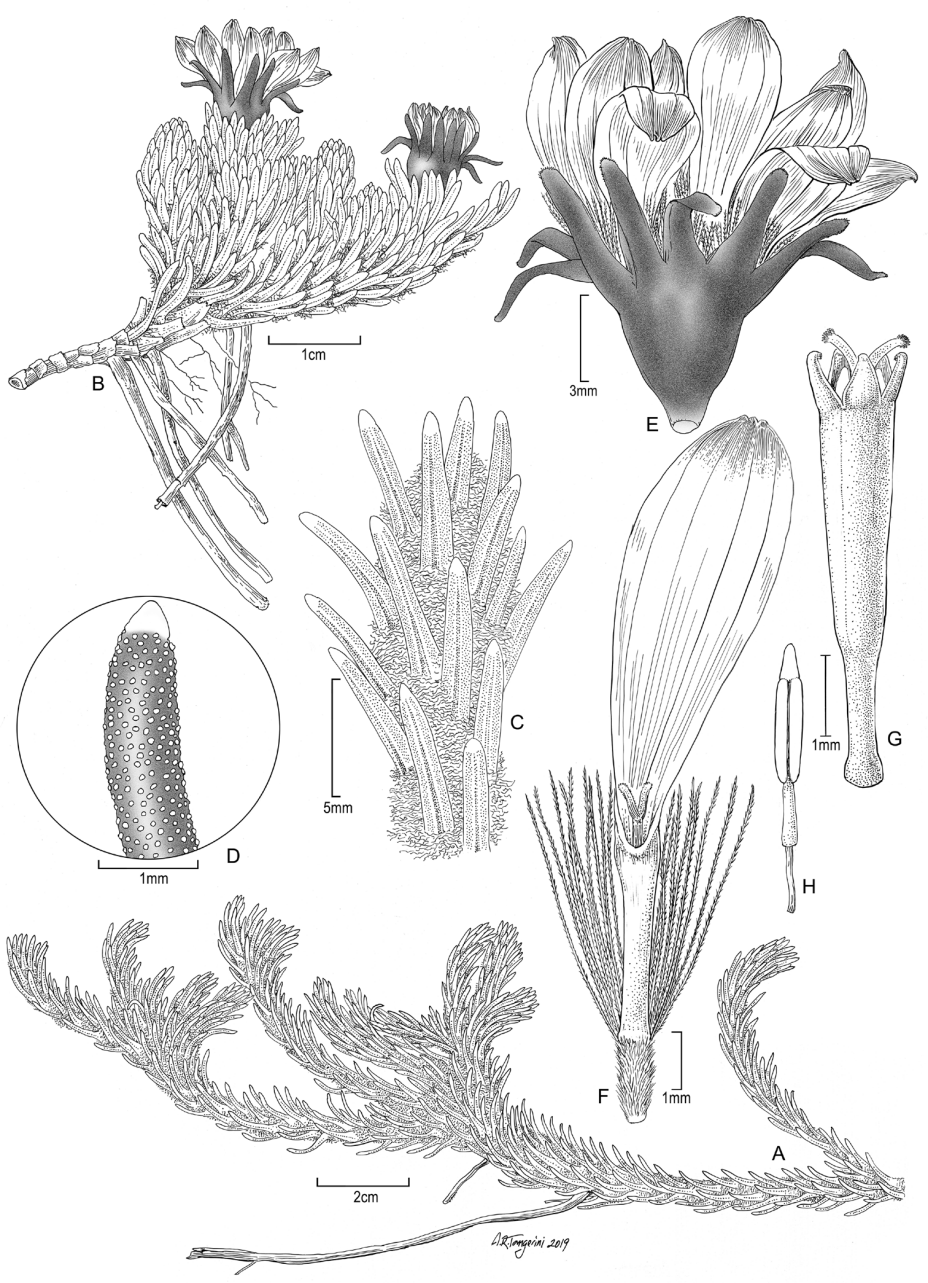
*Xenophyllumfunkianum***A** habit **B** habit with capitula **C** stem apical part **D** detail of leaf apex **E** capitulum **F** ray floret and achene (frontward bristles removed) **G** disc floret without pappus and achene **H** stamen. All details drawn from *Funk & Bonifacino 14059* (US). Illustration by Alice Tangerini.

**Figure 2.**
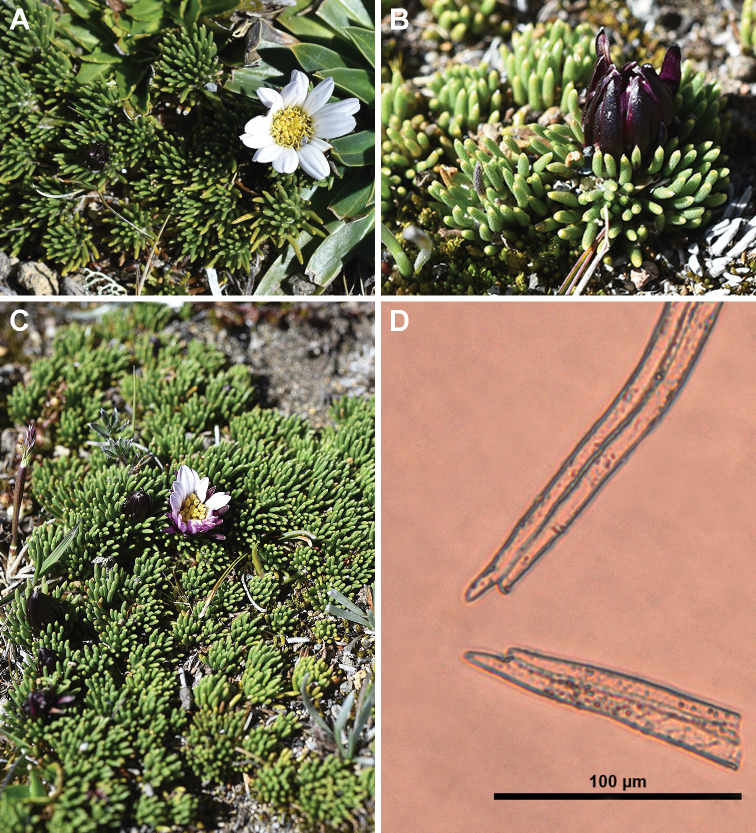
*Xenophyllumfunkianum***A** capitulum **B** involucre and leaves **C** habit **D** apex of achene trichomes (20× Standard 16WL); material taken from *Funk & Bonifacino 14059* (US).

#### Distribution and habitat.

**Figure 3.**
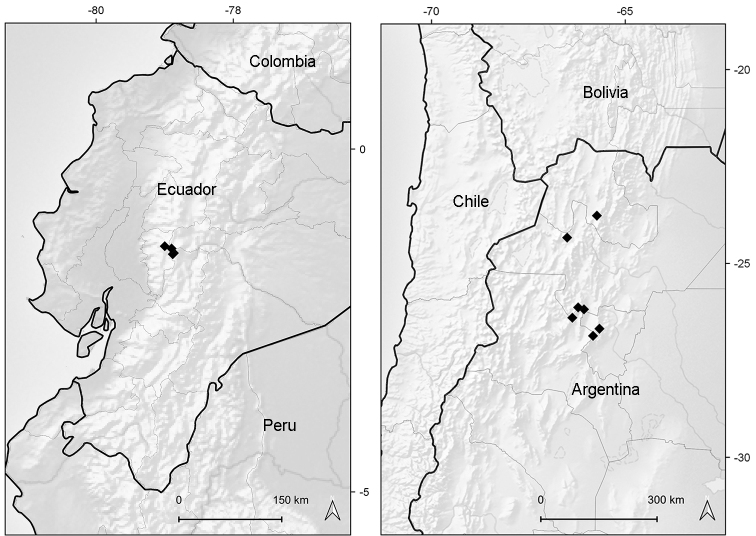
Distribution map of *Xenophyllumfunkianum* (left hand) and *X.lorochaqui* (right hand).

Endemic to Ecuador (provinces of Bolívar and Chimborazo) (Fig. 3). It grows in exposed places and sandy soils of the dry superparamo, at elevations of 4100–4300 m.

#### Phenology.

Flowering from April to July.

#### Etymology.

The epithet honors the American botanist Vicki A. Funk (1947–2019), who greatly contributed to the understanding of the family Compositae worldwide.

#### Discussion.

*Xenophyllymfunkianum* is morphologically close to *X.rigidum* (Kunth) V.A.Funk, a species distributed in central Ecuador which overlaps its distribution area. They can be easily differentiated by the leaf lamina size (5.3–7.8 × 0.8–0.9 mm in *X.funkianum* vs. 12.6–13.5 × 2.3–2.4 mm in *X.rigidum*), involucre size (9–11 × 5–7 mm in *X.funkianum* vs. 11.5–12.8 × 7.2–11.3 mm in *X.rigidum*), and number of disc florets (20–23 in *X.funkianum* vs. 38–41 in *X.rigidum*). Moreover, *X.rigidum* is a larger plant and the capitula are completely enclosed among the leaves. *Xenophyllymfunkianum* also shows morphological affinities with *X.humile* (Kunth) V.A.Funk; indeed, previous botanists confused the new species with it. They differ in the leaf shape (straight in *X.funkianum* vs. usually articulate in *X.humile*), involucre length (9–11 mm in *X.funkianum* vs. 4.6–9.3 mm in *X.humile*), and achene indumentum (white-villous in *X.funkianum* vs. glabrous in *X.humile*). Their way of growing is also different; *X.humile* forms dense mats or hummocks, whereas *X.funkianum* is rather a creeping plant. Another similar species is *X.roseum* (Hieron.) V.A.Funk, a species known from the Ecuadorian provinces of Azuay and Cañar that does not overlap the distribution area with the new species. Any confusion is unlikely since *X.roseum* displays pink ray florets and the young leaves usually bear a quickly deciduous arista up to 0.5 mm.

The white-villous achene indumentum of *X.funkianum* is composed of twin filiform trichomes, with acute to subacute, asymmetrical, slightly forked apex (Fig. 2D). This type of indumentum is also found in *X.rigidum* and *X.roseum*; however, most species of the genus have glabrous achenes.

#### Additional specimens examined (paratypes).

**Ecuador. Bolívar**: road to Salinas, 1.8 km W of Guaranda-Ambato hwy., 1°25'S, 79°0'W, 25 Jun 1989, *L.J. Dorr & I. Valdespino 6474* (QCA barcode 159734, QCNE-47994, US barcode 00622748); **Chimborazo**: Mt. Chimborazo area, side road ends and connects to trail that leads to the *Polylepis* forest, 1°32'S, 78°53'W, 20 Apr 2018, *V.A. Funk & J.M. Bonifacino 14061* (US); W side of the Chimborazo volcano, arenal around loma Guagua Lozán, 1°27'S, 78°54'W, 3 Jul 1999, *P. Sklenář 7528* (QCA barcode 161918, QCNE-159009, PRC n.v.).

### 2. Xenophyllum lorochaqui

Taxon classificationPlantaeAsteralesAsteraceae

J.Calvo & V.A.Funk sp. nov.

68F1018B-4CE5-5B3E-A9A7-744E14BD6776

urn:lsid:ipni.org:names:77204850-1

Fig. 4

#### Diagnosis.

*Xenophyllumlorochaqui* can be identified by the glabrous erect stems 10–20 cm tall, which usually only bear leaves on the upper part, leaf laminas 7.9–11.5 mm long, leaf apex 3-notched with the central lobe entire or barely notched and longer than the lateral ones, involucres with ca. 13 involucral bracts, and by displaying 26–39 ray florets with white corollas.

#### Type.

**Argentina.** Catamarca: El Cajón, Negroara, [26°24'S, 66°22'W], 15 Jan 1914, *L. Castillón 3365* (holotype: LIL-26677!; isotypes: BM s.n.!, BR s.n.!, US barcode 00622893!, W-334!).

#### Description.

Suffruticose plants, forming clumps of erect stems, 10–20 cm tall. ***Rhizomes*** 5–10 cm long, 0.6–0.8 cm in diam., horizontal to oblique, glabrous. ***Stems*** branched, glabrous, usually only bearing leaves on the upper part. ***Leaves*** simple, alternate, imbricate, prolonged into a sheath-like base glabrescent or with evanescent arachnoid trichomes; leaf laminas linear, broadened upward, 7.9–11.5 mm long, 2.3–2.7 mm wide, entire, 3-notched at the apex, with the central lobe longer than lateral ones, conduplicate upward in cross section, glabrous, unconspicuously nerved on both faces, fleshy, matte; central leaf lobe 1.0–1.6 mm long (lateral ones 0.5–0.8 mm long), 1.3–1.6 mm wide at the maximum width point, entire or notched, obtuse. ***Capitula*** radiate, solitary, terminal, erect, sessile. ***Involucres*** 10.3–10.9 mm long, 6.6–8.7 mm wide, cupuliform, with bracts fused at the base, glabrous; involucral bracts ca. 13, 5.9–7.0 mm long, 1.4–2.5 mm wide at the base, obtuse at the apex, greenish; without supplementary bracts. ***Ray florets*** 26–39, corollas 8.3–9.2 mm long, 1.1–1.4 mm wide, 4-veined, subentire to 3-toothed at the apex, conspicuously surpassing the involucre, white. ***Disc florets*** 40–57, corollas 4.2–5.5 mm long, 5-lobed, yellowish; style branches truncate with a crown of sweeping hairs, yellowish; anther bases auriculate; anther appendages two times longer than wide, ca. 0.5 × 0.2 mm. ***Achenes*** 2.5–3.1 mm long, 0.7–0.9 mm wide, cylindrical, 8–9-ribbed, glabrous; pappus 4.3–6.1 mm long, barbellate, whitish. Chromosome number unknown.

**Figure 4.**
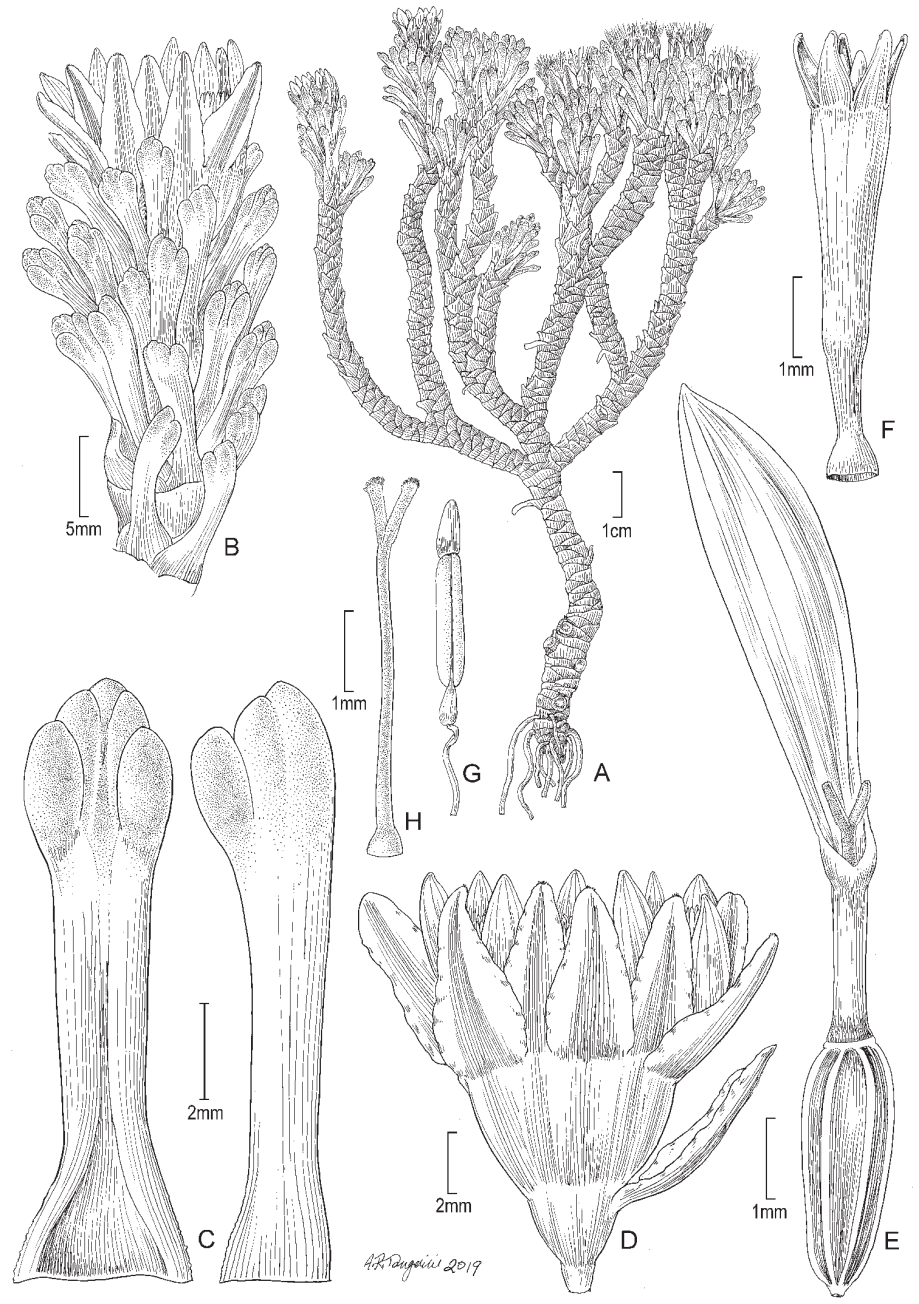
*Xenophyllumlorochaqui***A** habit **B** stem apical part with capitulum **C** adaxial leaf surface (left hand) and vertical leaf profile (right hand) **D** capitulum **E** ray floret with achene (pappus removed and not drawn) **F** disc floret without pappus and achene **G** stamen **H** style. **A, B** drawn from *Díaz s.n.* (GH) and **C–H** from *Castellanos s.n.* (BA). Illustration by Alice Tangerini.

#### Distribution and habitat.

Endemic to northwestern Argentina (provinces of Catamarca, Jujuy, Salta, and Tucumán) (Fig. 3). It grows in exposed rocky slopes and bare soils of the dry Puna ecoregion, between elevations of 3500‒5000 m.

#### Phenology.

Flowering from January to March.

#### Etymology.

The epithet *lorochaqui* is the vernacular name of this plant as it was stated on the labels of the collections made by the Argentinian botanist León Castillón (pupil of botanist Miguel Lillo). It means parrot’s foot referring to the leaf shape. The Spanish word “loro” means “parrot” and derives from “roro”, used by some Taíno peoples and presumably adopted by the Spaniards during the colonization. On the other hand, “chaqui” is a Quichuan word meaning “foot”.

#### Discussion.

*Xenophyllumlorochaqui* is morphologically close to *X.incisum* (Phil.) V.A.Funk, *X.dactylophyllum* (Sch.Bip.) V.A.Funk, and *X.poposa* (Phil.) V.A.Funk, and it partially overlaps its distribution area with *X.incisum* and *X.poposa*. The differences against *X.incisum* are the leaf lamina length (3.2–7.3 mm vs. 7.9–11.5 mm in *X.lorochaqui*), the length of the leaf apex lobes (similar vs. central lobe longer than lateral ones in *X.lorochaqui*), the involucre length (6.4–8.3 mm vs 10.3–10.9 mm in *X.lorochaqui*), the involucral bract number (ca. 8 vs. ca. 13 in *X.lorochaqui*), and the ray floret number (8–13 vs. 26–39 in *X.lorochaqui*). *Xenophyllumincisum*, moreover, is a species restricted to the banks of the salt lagoons and plains with a certain humidity of the desertic Puna ecoregion. With regard to *X.dactylophyllum*, the leaf apex shape let anyone readily discriminate from one another. In this latter species, it is ca. 9-divided (finger-like) with the primary division extending deeper than the subsequent ones, whereas in *X.lorochaqui* the leaf apex is 3-notched with the central lobe longer than lateral ones. *Xenophyllumdactylophyllum* is distributed from central Peru to central Bolivia. Some useful characters to differentiate the new species from *X.poposa* are the stem indumentum (arachnoid vs. glabrous in *X.lorochaqui*), the leaf lamina length (2.5–6.1 mm vs. 7.9–11.5 mm in *X.lorochaqui*), the involucral bract number (8–9 vs. ca. 13 in *X.lorochaqui*), and the ray floret number (8–11 vs. 26–39 in *X.lorochaqui*).

It is important to bring to light some considerations that previous botanists made regarding the identification of this species. The Argentinian D. Rodríguez identified it *in sched.* as “Werneriaincisa Phil. vel aff.!” (*Werneriaincisa* or similar [≡ *X.incisum*]), and annotated “f. foliis triplo longioribus” (leaves three times longer); see *Rodríguez 1382*. This latter remark perfectly matches one of the aforementioned characters that discriminates *X.lorochaqui* from *X.incisum*. On the other hand, L. Castillón identified his own collection as “Werneria dactylophylla Wedd. aff.”, which also reveals the failure of providing an accurate identification; see *Castillón 3365*. Later, Cabrera (1948) and Freire and Ariza-Espinar (2014) placed the collection *Rodríguez 1382* under the varietal name X.incisumvar.pubescens (Rockh.) Cabrera & S.E.Freire.

On an orthographic note, it is important to state that the epithets *lorochaqui* and *poposa* respond to the respective vernacular names of these species, and therefore, they are nouns in apposition not to be declined.

#### Additional specimens examined (paratypes).

**Argentina. Jujuy**: Tumbaya, cerro Moreno, 23°46'S, 65°44'W, 7 Feb 1929, *S. Venturi 9289* (US barcode 00622892); **Salta**: Cafayate, sierra de los Quilmes, 26°11'S, 66°4'W, 28 Jan 1943, *A. Castellanos s.n.* (BA-47088); abra del Gallo, ca. 40 km al SW de S. Antonio de los Cobres, en el camino a Pastos Grandes, 24°20'S, 66°30'W, 17 Dec 1946, *A. Krapovickas 3215* (LIL-433562, SI s.n.); nevado del Cajón [sierra de Quilmes], 26°8'S, 66°13'W, 1 Mar 1914, *D. Rodríguez 1382* (BA-25293, BR s.n.); **Tucumán**: cerro Muñoz, 26°52'S, 65°50'W, Jan 1916, *L. Castillón s.n.* (BR s.n. [mixed with *X.poposa*, individual on the bottom]); Tafí, cumbres de San José (La Mina), 26°41'S, 65°40'W, Mar 1933, *Díaz s.n.* (GH s.n., LIL-58643); Tafí, cumbre de Chasquivil, 26°41'S, 65°40'W, 12 Jan 1945, *D. Olea 252* (LIL-122530); Chicligasta, estancia Las Rosas, 15 Jan 1927, *S. Venturi 6342* (US barcode 00622889); Tafí, sierra del Cajón [sierra de Quilmes], Los Chuscos, 7 Feb 1926, *S. Venturi 6647* (US barcode 00622888).

## Supplementary Material

XML Treatment for Xenophyllum funkianum

XML Treatment for Xenophyllum lorochaqui
